# Supplementary material for: MetaRibo-Seq measures translation in microbiomes
Source: Nat Commun. 2020 Jun 29;11:3268. doi: 10.1038/s41467-020-17081-z (PMC7324362; doi:10.1038/s41467-020-17081-z)
Supplement: Supplementary file 10 — Supplementary Data 7 [file 41467_2020_17081_MOESM10_ESM.zip › File2/Confidence_VeryHigh_Taxonomy/329990_out.krona.html]

Javascript must be enabled to view this page.

members
magnitude
magnitudeUnassigned
count
unassigned
taxon
rank

329990\_out

12

12
2
superkingdom

12
1239
phylum

12
186801
class

order
1

SRS1055034\_contig\_number\_contig-100\_20773.20774
12
186802

1
family
31979

1
1485
genus


SRS1041145\_contig\_number\_2725
1
1262774
species

1
541000
family

1
216851
genus


SRS012273\_contig\_number\_9413
1
853
species

186803
1
9

SRS052697\_contig\_number\_14144
family

6
572511
genus


SRS022071\_contig\_number\_32988SRS104636\_contig\_number\_28438SRS147139\_contig\_number\_41462
3
1262757
species


SRS015431\_contig\_number\_80626SRS098644\_contig\_number\_25821SRS148721\_contig\_number\_56114
3
1896976
species

genus
841
1

species
360807
1

SRS149325\_contig\_number\_contig-100\_41591.41592

genus
1407607
1

1

SRS148159\_contig\_number\_contig-100\_3500.341509
species
1150298
